# Effect of Folic Acid Supplements on Progesterone Profile and Blood Metabolites of Heat-Stressed Holstein Cows during the Early Stage of Pregnancy

**DOI:** 10.3390/ani12151872

**Published:** 2022-07-22

**Authors:** Abdelrahman A. Kilany, Abdel-Halim A. El-Darawany, Akram A. El-Tarabany, Khaled M. Al-Marakby

**Affiliations:** 1 Radioisotopes Applications Division, Department of Biological Applications, NRC, Egyptian Atomic Energy Authority, Inshas, Cairo P.O. Box 13759, Egypt; elkelany_86@icloud.com; 2 Department of Animal Production, Faculty of Agriculture, Zagazig University, Zagazig P.O. Box 44511, Egypt; a.a.eldarawany@hotmail.com (A.-H.A.E.-D.); jnakhaled303@yahoo.com (K.M.A.-M.)

**Keywords:** fertility, Holstein, folic acid, pregnancy

## Abstract

**Simple Summary:**

The fertility of a dairy cow can be described as the female’s ability to conceive and maintain pregnancy when insemination is performed at the proper time relative to ovulation. In this context, poor estrous detection and embryonic or fetal losses are common causes for reduced reproductive performance in dairy cows. Furthermore, heat stress severely deteriorates conception and pregnancy rates in dairy farms. The aim of this study was to see how oral folic acid (FA) supplements affect progesterone levels, blood metabolites, endocrine patterns, and blood biochemical concentrations in heat-stressed pregnant cows. Oral FA supplementation (10 μg kg^−1^) in the first month of gestation improved the progesterone profile, as well as blood folates, pregnancy associated glycoprotein (PAG), growth hormone (GH), and Insulin-like growth factor-1 (IGF-1) concentrations in heat-stressed Holstein cows.

**Abstract:**

The aim was to elucidate the impact of oral folic acid (FA) supplements on progesterone profile, blood metabolites and biochemical indices of heat-stressed Holstein cows during the early stage of pregnancy. The study lasted from the day of artificial insemination through the end of the fourth week of pregnancy. The first group (CON, *n* = 17) received 0 μg of FA/kg BW as a control. The second and third groups received oral FA doses of 5 (FA_5_, *n* = 19) and 10 (FA_10_, *n* = 20) μg kg^−1^ BW, respectively. At the 2nd and 3rd weeks of pregnancy, the FA_10_ group had greater progesterone levels than the CON group (*p* < 0.05). The FA_10_ group had a greater progesterone level than the FA_5_ and CON groups at the fourth week of pregnancy (*p* < 0.01). The FA_10_ group had higher folate levels than CON group during the first three weeks of pregnancy (*p* < 0.01). Both FA-supplemented groups had significantly greater serum folates than the CON group by the end of the fourth week of pregnancy (*p* < 0.01). At the 2nd and 4th weeks of pregnancy, the FA_10_ group had greater levels of serum glucose and globulin than the CON group (*p* = 0.028 and 0.049, respectively). Both FA-supplemented groups had greater serum growth hormone (GH) levels at the 4th week of pregnancy (*p* = 0.020). Additionally, the FA_10_ group showed significantly higher levels of IGF-1 at the 2nd and 4th week of gestation (*p* = 0.040 and 0.001, respectively). FA supplementation decreased the levels of non-esterified fatty acid (NEFA) at the 2nd and 4th week of gestation (*p* = 0.020 and 0.035, respectively). Additionally, the FA_10_ group showed significantly higher pregnancy-associated glycoprotein (PAG) levels at the 2nd and 4th week of gestation (*p* = 0.005 and 0.001, respectively). In conclusion, oral FA supplementation (10 mcg kg^−1^) in the first month of gestation improved the progesterone profile, as well as blood folates, PAG, GH, and IGF-1 concentrations in heat-stressed Holstein cows. These findings could be useful in developing practical strategies to keep dairy cows’ regular reproductive patterns under heat stress conditions.

## 1. Introduction

The fertility of dairy cows can be described as the female’s ability to conceive and maintain pregnancy when insemination is performed at the proper time relative to ovulation [1]. In this context, poor estrous detection and embryonic or fetal losses are common causes for reduced reproductive performance in dairy cows [2]. Furthermore, heat stress severely deteriorates the conception and pregnancy rates in dairy farms [3]. This can be attributed to the negative effects of hyperthermia on the biological functions of female reproductive tract [4]. Jordan [5] claimed that the unfavorable effects of thermal stress could be observed from day 42 before insemination to day 40 after insemination. Furthermore, the influence of maternal heat stress on embryos varies depending on the stage of development; nonetheless, the initial days after fertilization are the most essential for the embryo [6].

Folic acid (FA) is a B-complex vitamin with the sole biochemical role of mediating the transfer of 1-C units in animals [7]. FA also has a variety of activities, including regulating neurotransmission and gene expression [8,9]. Folate metabolism appears to have a significant impact on the synthesis of milk proteins in the epithelial cells of mammary glands [10,11]. Although rumen microorganisms can generate folates, it has not been proven that such amounts are sufficient for dairy cows to perform at their best [12]. To some extent, the forage:concentrate ratio may influence the amount of folates produced in the rumen [13]. In this context, rumen breakdown of orally supplied FA appears to be very high, as FA supplement hardly raises folate concentrations in the digesta [14]. Total serum folates in dairy cows declined by 40% from two months postpartum until parturition [15]. Additionally, supplemental FA in gestation and lactation enhanced milk yield and milk protein yields in multiparous cows but not in primiparous cows [16].

It is widely acknowledged that a successful pregnancy is the most crucial aspect in ensuring a profitable dairy cow. In this regard, FA has attracted a lot of interest in animal research, especially during pregnancy. In multiparous dairy cows, Duplessis et al. [17] found that FA supplementation improved reproduction indices. The use of a periconceptional FA supplement slightly increased the number of twin pregnancies in humans [18]. Only a small number of studies have looked into the impact of FA supplements on the reproductive performance of heat-stressed cows [9,19], to the best of our knowledge. As a result, the aim of this study was to see how oral FA supplements affect progesterone levels, blood metabolites, and blood biochemical concentrations in heat-stressed pregnant cows.

## 2. Materials and Methods

The current research was performed at dairy station number 2 (30°36′16.0″ N, 31°54′21.5″ E), El Salhiya company for investment and development, New Salhiya, Sharkia governorate, Egypt.

### 2.1. Animals, Management and Experimental Design

In total, 120 multiparous cows (4–8 years of age) from El Salhiya Company for investment and development were used in the first stage of this experiment. The selected cows had homogenous body condition scores (3.4 ± 0.2). All cows were regularly monitored during the early post-partum period to exclude the reproductive disorders and then vaccinated against diseases that impede the reproductive traits by veterinarians. The cows were equally divided into three groups (each 40 cows) and housed in three yards (50 m × 60 m) with open stalls. The animals were fed a total mixed ration (TMR). The TMR was adjusted to achieve the optimal requirements of nutrients for milk production and body condition [20]. The chemical composition of TMR was described in Table 1. All cows were milked twice daily at 12 h intervals. The first group received 0 μg of folic acid/kg BW and served as control (CON). Orally, the second and third groups received 5 (FA_5_) and 10 (FA_10_) μg of folic acid/kg BW two times weekly, respectively. The folic acid tablets (Folate 1.000 μg folic acid) were purchased from Nutricost Co., Vineyard, UT, USA. The tablets were administered using a long cylindrical bolus gun, which is a tube made of stainless steel that has a spring-action handle. The experiment started at the day of artificial insemination (AI) to the end of fourth week of gestation period. When pregnancy was confirmed at day 28 following the AI, only cows that conceived were included in this study, and hence the actual sizes of the experimental groups were 17, 18 and 20 cows in CON, FA5 and FA10 groups, respectively.

### 2.2. Reproductive Management and Experimental Procedures

During the first two weeks post partum, all cows having an abnormal puerperium period were eliminated from the study (dystocia, twinning, retained placenta, ovarian cysts, primary metritis or ketonuria). Cows with an average body condition score of 3.5 ± 0.3 were considered to be in a good condition. The Ovsynch synchronization protocol was initiated at 45 ± 4 day postpartum in all groups. All cows were given 10 g GnRH (day 0; Buserelin; Receptal; Intervet), 25 mg PGF2α (day 7; dinoprost; Lutylase; pfizer), and one dosage of PGF2α 56 h before the second dose of GnRH. Artificial insemination (AI) was conducted 18 h following the second dosage of GnRH. The insemination processes were practiced by three expert technicians. Diagnosis of pregnancy was conducted 28 days following the AI (transrectal ultrasonography; Pie Medical, Maastricht, The Netherlands), and only cows that conceived to AI were included in this study (CON, *n* = 17; FA_5_, *n* = 19; FA_10_, *n* = 20). Hence, the samples from those pregnant animals were included in the current study.

### 2.3. Estimation of Progesterone Profile and Serum Folates

On a weekly basis, blood (3 mL) was sampled from the jugular vein for four consecutive weeks to evaluate the concentrations of progesterone and folates. Centrifugation at 1008× *g* for 15 min separated the serum, which was then kept at −20 °C. The serum progesterone levels were determined using a radioimmunoassay method (commercial kits, Diagnostic Product Corporation, Los Angeles, CA, USA). In antibody-coated tubes, unknown samples or standards are treated with I125-labeled hormone. The liquid contents of the tube were aspirated after incubation, and the radioactivity was measured in a computerized gamma counter at the Egyptian Atomic Energy Authority, Nuclear Research Centre. The intra- and inter-assay coefficients of variation were 4.9 and 7.5 percent, respectively. The concentrations of serum folic acid were determined using chemical commercial kits and quantitative enzymatic colorimetric techniques (MDSS GmbH, Hannover, Germany).

### 2.4. Estimation of Blood Metabolites and Biochemical Indices

Blood was taken from the jugular vein at the end of the second and fourth weeks of pregnancy to assess the concentrations of various blood metabolites and biochemical markers. Centrifugation at 1008× *g* for 15 min was performed to separate the serum, which was then frozen and stored at −20 °C until analysis. Total protein, urea, triglycerides, cholesterol, glucose, and albumin concentrations were determined using chemical commercial kits and quantitative enzymatic colorimetric techniques (MDSS GmbH, Hannover, Germany). The concentration of pregnancy associated glycoprotein (PAG) was detected by enzyme-linked immune sorbent assay (ELIZA) technique, with Bovine PAG antibody (Bioassay technology laboratory Co, Ningguo, Shanghai, China, Cat No MBS1602347), and the standard curve range was 0.05 ng/mL–20 ng/mL, sensitivity 0.02 ng/mL. The levels of serum non-esterified fatty acid (NEFA) were measured by ELIZA technique with bovine NEFA antibody (Bioassay technology laboratory Co, Ningguo, Shanghai, China, Cat No E0021Bo), with a standard curve range of 2 μmol/L–600 μmol/L and a sensitivity of 1.17 μmol/L. The concentrations of Beta-Hydroxybutric Acid (BHB) and insulin-like growth factor-1 (IGF-1) were determined also by ELIZA technique (Boster Biomedical Technology, Valley Ave, Pleasanton, CA, USA). The concentration of serum cortisol was determined (Diagnostic Product Corporation, Los Angeles, CA, USA).

### 2.5. Metrological Data

The temperature–humidity index indicates heat stress by representing the interaction impact of both temperature and humidity. The records of daily temperature and humidity during this study were procured from the New Salhiya metrological station, approximately 9 km away from the farm area. Values of the THI were calculated as follows [21]:

THI=[(1.8×AT)+32]−[0.55−(0.0055×RH)]×[(1.8×AT)−26]

where AT is ambient temperature (°C) and RH is relative humidity (%). The THI is considered to be high when the value exceeds 75 (Table 2).

### 2.6. Statistical Analysis

The General Linear Model procedures (GLM) of the IBM SPSS software application were used to examine the data (Version 16.0; IBM Corp., Armonk, NY, USA). For blood metabolites and biochemical indices (2nd and 4th week), the statistical model was constructed as follows:

$$Y_{\mathrm{ijk}}=\mu +T_{i}+P_{j}+e_{\mathrm{ijk}}$$

where:

Y_ijk_: An observation of each trait.
μ: The overall mean.
T_i_: The fixed effect of treatment ith (1, 2, 3).
P_j_: The fixed effect of parity jth.
e_ijk_: The random effect of error.

Data repeatedly measured (progesterone profile and serum folates) were subjected to ANOVA with repeated measures (IBM SPSS software). Duncan’s Multiple Range Test was used to compare the means. Significance was stated at *p* < 0.05.

## 3. Results

### 3.1. Progesterone Profile and Serum Folates

**Figure 1:**
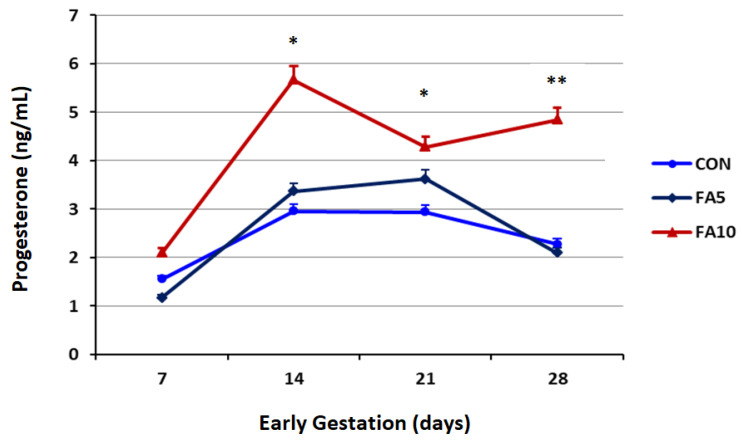
Effects of folic acid supplements (FA_5_ and FA_10_) on progesterone profile of heat-stressed Holstein cows during early gestation period. The concentrations of progesterone were affected by treatment (*p* < 0.001) and time (*p* = 0.003), but not treatment by time (*p* = 0.315). *, ** Significant differences at *p* < 0.05 and 0.01, respectively.

The effects of FA supplements on progesterone profile during the early stage of pregnancy in heat-stressed Holstein cows are presented in Figure 1. The oral FA supplements significantly improved the serum progesterone level (*p* < 0.001). Throughout the first week of pregnancy, there were no significant differences between the experimental groups. Meanwhile, the FA_10_ group had significantly greater progesterone levels than the CON group at the 2nd and 3rd weeks of pregnancy (*p* < 0.05). By the end of the fourth week of pregnancy, the FA_10_ group had significantly higher progesterone levels than the FA_5_ and CON groups (*p* < 0.01).

**Figure 2:**
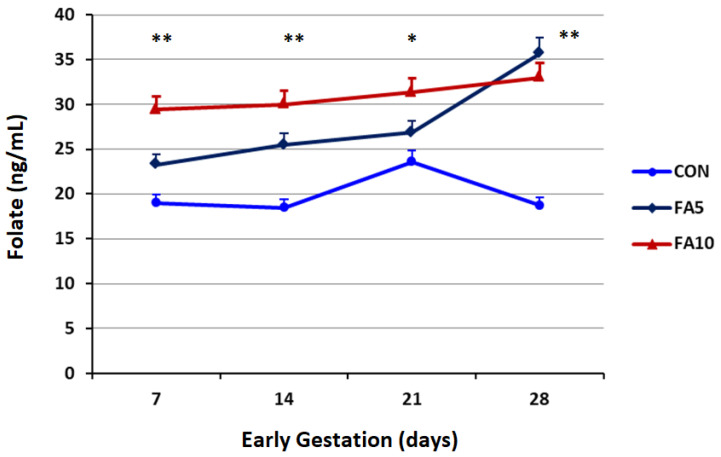
Effect of folic acid supplements (FA_5_ and FA_10_) on serum folate profile in heat-stressed Holstein cows during the early gestation period. The concentration of folate was affected by treatment (*p* = 0.009) and time (*p* = 0.002), but not treatment by time (*p* = 0.147). *, ** Significant differences at *p* < 0.05 and 0.01, respectively.

The effects of FA supplements on the levels of serum folates during the early stage of pregnancy in heat-stressed Holstein cows are shown in Figure 2. The concentrations of serum folates were significantly affected by FA supplements (*p* = 0.009). During the first three weeks of gestation, the FA_10_ group exhibited significantly higher folates level than the CON group (*p* < 0.01). Both FA-supplemented groups had significantly greater serum folates than the CON group by the end of the fourth week of pregnancy (*p* < 0.01).

### 3.2. Blood Metabolites and Biochemical Indices

Table 3 shows the impact of FA supplementation on blood metabolite concentrations in heat-stressed Holstein cows. At the 2nd and 4th weeks of pregnancy, the FA_10_ group had greater levels of serum glucose and globulin than the CON group (*p* = 0.028 and 0.049, respectively). When compared to the CON group, FA supplements significantly reduced serum urea at the 4th week of pregnancy (*p* = 0.028). Meanwhile, there were no significant differences in total protein, cholesterol, triglycerides, HDL, and LDL concentrations across the experimental groups.

Table 4 shows the effects of FA supplementation on cortisol, GH, and IGF-1 levels in heat-stressed Holstein cows during the early stage of pregnancy. Although cortisol levels did not change significantly, both FA-supplemented groups had significantly greater levels of GH than the CON group at the 4th week of pregnancy (*p* = 0.020). Additionally, the FA_10_ group had significantly higher IGF-1 levels at the 2nd and 4th week of gestation (*p* = 0.040 and 0.001, respectively).

Table 5 shows the impacts of FA supplementation on NEFA, BHB, and PAG levels in heat-stressed Holstein cows during the early stage of pregnancy. FA supplements significantly decreased the levels of NEFA at the 2nd and 4th week of gestation (*p* = 0.020 and 0.035, respectively). Meanwhile, there were no significant changes in BHB concentrations at the 4th week of pregnancy (*p* = 0.069). At the 2nd and 4th week of gestation, the FA_10_ group had significantly higher PAG levels compared to the FA_5_ and CON groups (*p* = 0.005 and 0.001, respectively).

## 4. Discussion

The purpose of this study was to see how FA supplements affected the progesterone profile and blood chemistry of heat-stressed Holstein cows during the first month of pregnancy. Progesterone is thought to have an important role in the implantation and growth of embryos in the early stages of pregnancy [22]. Furthermore, cows exposed to summer heat stress often have a considerable reduction in progesterone levels [23]. This could be attributed to decreased progesterone production or perhaps to disruption of corpus luteum (CL) development during thermal stress [24]. Herein, FA supplements (10 mcg kg^−1^) significantly improved the progesterone profile in heat-stressed cows during the first month of gestation. Hence, this may enhance the implantation process of embryos under chronic thermal stress. El-Tarabany et al. [22] suggested that FA supplements considerably increased serum progesterone concentrations throughout the early stages of pregnancy, which is in line with our findings. In this regard, Starbuck et al. [25] found a positive relationship between plasma progesterone levels and pregnancy maintenance at week five of pregnancy. In heat-stressed mice, FA supplementation scavenges the reactive oxygen species in embryos and improves the cell number in blastocyst [26]. Shin and Shiota [27] also suggested that FA supplements decreased the incidence of neural tube abnormalities in heat-stressed mouse embryos. Furthermore, at high levels of THI, using progesterone- releasing intra-vaginal inserts (CIDRsynch protocol) maintains an acceptable and consistent pregnancy rate [3]. On day 16, uterine luminal concentrations of interferon tau were associated with progesterone levels on days 4 and 5, preventing luteolisis and thereby embryonic losses in pregnant cows [28].

Previous research has shown that the serum folate levels in dairy cows reduced by 40% from the second month post partum (around AI) through parturition [15], which is usually independent of an exogenous FA supplements [29]. Accordingly, ruminal synthesis of folates appears to be insufficient to restore serum folates throughout early pregnancy and lactation. In this context, the drastic decline of serum folates in multiparous dairy cows could reflect an elevated requirement for FA by the maternal–fetal complex. In the current study, the FA_10_ group exhibited significantly greater progesterone levels than the control group during the first three weeks of gestation. Both FA-supplemented groups had significantly higher levels of serum folates than the CON group by the end of the fourth week of pregnancy. Li et al. [30] found that serum folate concentrations increased linearly with increasing rumen protected FA (RPFA) supplementation in multiparous Holstein cows, which is consistent with our findings. Similarly, supplemental FA at 2.6 g/day significantly improved plasma folate concentrations [31]. Girard and Matte [16] also claimed that FA supplementation enhanced the concentrations of serum folate, which peaked in supplemented cows during the early lactation period.

Glucose, as the principal metabolic fuel for maintenance, fetal growth, and milk production, is an essential component of dairy cows’ protection against negative energy balance [32]. Due to a negative energy balance after calving, which is reflected by changes in blood metabolic and hormone profiles, high rates of body condition score reductions occur [33]. The large energy demand of dairy cows is partially supplied by gluconeogenesis, and glucose plays a vital role in metabolism and homeostasis [34]. In the current study, FA supplementation (FA_10_) increased the serum glucose in heat-stressed cows during the early gestation period. Consistent with these findings, Li et al. [30] observed significant increases in plasma glucose levels when multiparous dairy cows were fed RPFA-supplemented diets. Similarly, supplementing nursing cows with FA and vitamin B12 improves metabolic efficiency by raising plasma glucose or glucose irreversible loss rate in the early stages of lactation [31]. They discovered that amino acid gluconeogenesis, such as glycine, serine, threonine, and total sulphur amino acids, can promote serum glucose level. In a more recent study, Li et al. [35] found that adding RPFA to maternal diets boosted serum glucose levels in growing lambs. Duplessis et al. [36] also demonstrated that weekly intramuscular injections of FA raised plasma glucose in postpartum dairy cows (320 mg). The discrepancy in the results could be explained by the fact that the current study used a lower dose of FA than Duplessis et al. [36]. When compared to the CON group, FA supplementation reduced serum urea levels at the 4th week of pregnancy. In Holstein cows, Rastani et al. [37] observed that the concentration of urea depends on the DM intake and the variation of urea may be influenced by the levels of NEFA. Meanwhile, others reported that the RPFA supplements did not affect the levels of serum urea in growing lambs [35]. They also support our findings that FA supplements did not affect the levels of serum triglycerides and cholesterol.

The catabolic and anabolic activities of lipid in the animal body are reflected in serum lipid metabolites. In this scenario, negative energy balance triggers fat mobilization, resulting in a rise in blood NEFA and BHB concentrations [38]. The reduced serum NEFA and BHB in FA-supplemented groups (mostly FA10) may imply that body fat catabolism was dramatically reduced in heat-stressed pregnant cows. As a result of these findings, FA supplements improved energy balance and lowered body fat mobilization, indicating positive changes in BW of pregnant cows. Li et al. [30] observed that during the early lactation stage of Holstein cows, blood concentrations of NEFA and BHB dropped linearly as RPFA supplementation increased. Meanwhile, others suggested that serum NEFA and BHB were not influenced by the addition of RPFA to the diets of growing Hu lambs [35].

IGF-1 and GH play important roles in cell metabolism, adipogenesis, glucose and energy metabolism [39]. Hence, IGF-1 and GH are useful indicators for assessing the metabolic condition of animals. In the present study, both FA-supplemented groups exhibited significantly higher levels of GH anf IGF-1 at the 4th week of gestation. Similarly, the addition of RPFA to the lamb diets increased the levels of serum GH and IGF-1 at days 120 and 180 of age [35]. In this context, the increased levels of serum GH and IGF-1 in the RPFA-supplemented groups may be attributed to the elevated ruminal VFA concentrations or dietary protein availability [40,41]. Wang et al. [42] found that dietary FA supplementation raised IGF-1 levels at the 90th day of pregnancy in multiparous pregnant ewes. Nonetheless, they revealed that dietary FA supplementation had no effect on GH concentrations. In dairy heifers, weekly FA injections (40 mg) had no effect on serum GH and IGF-1 secretion [43].

The placenta of ruminants such as cows, ewes, and goats expresses a vast family of inactive aspartic proteinases known as PAG [44]. Furthermore, PAG concentration is a valid marker for placental growth and function that could be utilized to predict embryonic loss [45]. The FA_10_ group had considerably greater PAG levels than the FA_5_ and CON groups throughout the first month of pregnancy. This seems to be correlated with the higher progesterone levels in the FA_10_ group. Ayad et al. [46] found a favorable association between progesterone and PAG concentrations in dairy cattle during the first three months of pregnancy, which supports our findings. In this context, Ricci et al. [47] claimed that average PAG concentrations in dairy cows increase from day 15 to day 35 of pregnancy. During the time from day 29 to day 36 following AI, Nanas et al. [48] reported no variation in PAG levels between summer and winter pregnancies.

## 5. Conclusions

Finally, FA supplements (10 μg kg^−1^) given to heat-stressed Holstein cows during the first month of pregnancy enhanced their progesterone profile, as well as serum folates, PAG, GH, and IGF-1 levels. In heat-stressed pregnant cows, FA supplementation decreased NEFA and BHB levels. These findings could be useful in developing practical strategies for dairy cows to maintain their regular reproductive patterns under heat stress conditions.

## Figures and Tables

**Table 1:** Chemical composition of the total mixed ration (TMR).

Item	Dry Matter Basis (%)
Crude Protein	17.96
NDF	26.32
Cellulose	14.93
Hemicellulose	8.78
Lignin	2.62
Crude Fat	5.1
Ash	10.72
TDN	71.74
DE (Mcal/kg)	3.23
ME (Mcal/kg	2.58

TDN: total digestible nutrients; NDF: neutral detergent fiber; DE: digestible energy; ME: metabolizable energy.

**Table 2:** Average air temperature, humidity, and temperature humidity index (THI) during the early gestation period.

Experimental Weeks	Air Temperature, °C	Humidity, %	THI
1st week	37	75	81
2nd week	37	73	82
3rd week	36	76	81
4th week	36	75	80

**Table 3:** Effect of folic acid supplements on the concentrations of blood metabolites in heat-stressed Holstein cows during the early stage of pregnancy.

Parameter	Gestation (Week)	Experimental Groups				
		^1^ CON	^2^ FA_5_	^3^ FA_10_	^4^ SEM	*p*-Value
Glucose, mmol/L	2nd week	2.46 ^b^	2.35 ^b^	2.90 ^a^	0.18	0.041
	4th week	2.55	2.16	2.22	0.16	0.118
Urea, mmol/L	2nd week	4.52	4.85	4.46	0.28	0.856
	4th week	5.21 ^a^	4.17 ^b^	4.45 ^ab^	0.33	0.028
Total protein, g/dL	2nd week	7.27	6.73	5.91	0.75	0.622
	4th week	4.44	6.56	6.97	0.67	0.087
Albumin, g/dL	2nd week	4.56 ^a^	3.96 ^a^	2.16 ^b^	0.25	0.010
	4th week	3.65	3.29	4.57	0.32	0.134
Globulin, g/dL	2nd week	3.33	2.56	2.88	0.27	0.404
	4th week	3.28 ^b^	2.99 ^b^	4.20 ^a^	0.23	0.049
Cholesterol, mmol/L	2nd week	8.63	8.99	9.12	0.36	0.496
	4th week	8.54	9.14	8.06	0.32	0.405
Triglycerides, mmol/L	2nd week	5.31	6.38	5.36	0.32	0.095
	4th week	6.97	6.57	6.27	0.34	0.677
^5^ HDL, mmol/L	2nd week	4.06	2.61	4.24	0.22	0.053
	4th week	3.95	3.72	5.68	0.28	0.209
^6^ LDL, mmol/L	2nd week	3.30	3.51	2.90	0.17	0.488
	4th week	3.09	3.11	2.22	0.15	0.404

^1^ Control group; ^2^ folic acid supplements 5 μg kg^−1^; ^3^ folic acid supplements 10 μg kg^−1^; ^4^ standard error of mean; ^5^ high-density lipoprotein; ^6^ low-density lipoprotein. ^a,b^ Values within a row with different superscripts differ significantly.

**Table 4:** Effect of folic acid supplements on the concentrations of cortisol, GH and IGF-1 in heat-stressed Holstein cows during the early stage of pregnancy.

Parameter	Gestation (Week)	Experimental Groups				
		^1^ CON	^2^ FA_5_	^3^ FA_10_	^4^ SEM	*p*-Value
Cortisol, μg/dL	2nd week	4.13	3.20	3.26	0.31	0.189
	4th week	3.92	3.16	4.29	0.24	0.323
^5^ GH, pg/mL	2nd week	0.280	0.250	0.254	0.040	0.889
	4th week	0.220 ^b^	0.310 ^a^	0.303 ^a^	0.033	0.020
^6^ IGF-1, pg/mL	2nd week	396 ^b^	301 ^b^	786 ^a^	30.4	0.040
	4th week	349 ^c^	486 ^b^	729 ^a^	31.7	0.001

^1^ Control group; ^2^ folic acid supplements 5 μg kg^−1^; ^3^ folic acid supplements 10 μg kg^−1^; ^4^ standard error of mean; ^5^ growth hormone; ^6^ insulin-like growth factor-1. ^a,b,c^ Values within a row with different superscripts differ significantly.

**Table 5:** Effect of folic acid supplements on the concentrations of NEFA, BHB and PAG in heat-stressed Holstein cows during the early stage of pregnancy.

Parameter	Gestation (Week)	Experimental Groups				
		^1^ CON	^2^ FA_5_	^3^ FA_10_	^4^ SEM	*p*-Value
^5^ NEFA, μmol/L	2nd week	173.5 ^a^	130.5 ^b^	109.0 ^b^	6.92	0.020
	4th week	100.9 ^a^	96.3 ^ab^	82.2 ^b^	4.15	0.035
^6^ BHB, mmol/L	2nd week	0.535 ^a^	0.405 ^ab^	0.377 ^b^	0.04	0.013
	4th week	0.349	0.379	0.374	0.03	0.069
^7^ PAG, ng/mL	2nd week	1.82 ^b^	2.42 ^b^	5.21 ^a^	0.19	0.005
	4th week	3.23 ^b^	2.44 ^b^	5.93 ^a^	0.18	0.001

^1^ Control group; ^2^ folic acid supplements 5 μg kg^−1^; ^3^ folic acid supplements 10 μg kg^−1^; ^4^ standard error. ^5^ Non- esterified fatty acid, ^6^ Beta-Hydroxybutric Acid, ^7^ pregnancy-associated glycoprotein. ^a,b^ Values within a row with different superscripts differ significantly.

## Data Availability

Data available on reasonable request from the corresponding author.
